# Advertising and Eco-Labels as Influencers of Eco-Consumer Attitudes and Awareness—Case Study of Ecuador

**DOI:** 10.3390/foods13020228

**Published:** 2024-01-11

**Authors:** Nelson Carrión-Bósquez, Iván Veas-González, Franklin Naranjo-Armijo, Mary Llamo-Burga, Oscar Ortiz-Regalado, Wilfredo Ruiz-García, Wilson Guerra-Regalado, Cristian Vidal-Silva

**Affiliations:** 1 Departamento de Administración, Facultad de Economía y Administración, Universidad Católica del Norte, Antofagasta 1270709, Chile; iveas@ucn.cl; 2 Carrera de Administración de Empresas, Instituto Superior Tecnológico Japón, Santo Domingo 170120, Ecuador; fnaranjo@itsjapon.edu.ec; 3 Escuela Profesional de Ingeniería en Agronegocios, Universidad Nacional de Cajamarca, Cajamarca 06001, Peru; mllamob@unc.edu.pe (M.L.-B.); oortizr@unc.edu.pe (O.O.-R.); wruizg@unc.edu.pe (W.R.-G.); 4 CENTRUM Catolica Graduate Business School, Pontificia Universidad Católica del Perú, Lima 15088, Peru; wguerrar@pucp.edu.pe; 5 Escuela de Computación e Informática, Facultad de Ingeniería, Ciencia y Tecnología, Universidad Bernardo O’Higgins, Santiago 8370993, Chile; cristianvidal@docente.ubo.cl

**Keywords:** green advertising, eco-labels, environmental attitudes, environmental awareness, green purchase intention

## Abstract

This study examined the impact of green advertising and eco-labels on the attitudes and environmental awareness of millennials purchasing eco-friendly products in shopping centers across Ecuador. The research utilized a quantitative, correlational, cross-sectional methodology with 430 millennials participating. A 20-item survey was administered face-to-face at shopping centers in Quito and Guayaquil, Ecuador. The validity of the research model was established through Confirmatory Factor Analysis (CFA) and Structural Equation Modeling (SEM), employing SPSS 20 and AMOS 24 for statistical evaluations. Findings reveal that green advertising significantly shapes environmental attitudes (β: 0.245) and awareness (β: 0.110), as well as directly influences the purchasing behavior (β: 0.154) towards green products. While eco-labels do not exert a direct effect on purchasing behavior (β: 0.128), they significantly inform attitudes (β: 0.406) and ecological awareness (β: 0.277) of millennials who purchase organic products. This paper is among the pioneering research to delineate the correlation between green advertising elements and the purchasing patterns of green products among millennials in a developing nation. It concludes that marketing strategies centered on green advertising and eco-labels do affect millennials’ attitudes and environmental consciousness, but only advertising has a direct impact on purchasing behaviors, contrary to eco-labels. The research bears social significance as it affirms that millennials are attentive to environmental issues and are actively engaged in promoting sustainability.

## 1. Introduction

The excessive consumption of products has generated growing concern due to its direct impact on environmental pollution [1]. This situation has highlighted the importance of adopting responsible consumption practices [2]. By choosing organic products and adopting more sustainable habits, consumers can significantly contribute to reducing the negative environmental impact in the world [3,4]. This transition towards more conscious and environmentally friendly consumption not only helps preserve natural resources but also promotes a healthier and more sustainable lifestyle for future generations [5,6].

Academic literature often employs terms like “green consumption”, “adoption of ecological or organic products”, and “green purchasing” to characterize consumer behaviors that are in harmony with environmental conservation [2,7]. Green consumption embodies a mindset and awareness that prioritize environmental well-being [3]. According to Liobikiene and Bernatoniene [8], such consumption transcends mere reduction in product acquisition; its primary aim is to diminish pollution. Research over time concurs that environmental concerns have galvanized consumer support for green consumption [2,5,9,10,11], thereby escalating demand for eco-friendly products. Consequently, modern businesses are compelled to respond to the competitive pressures of the market by adopting more sustainable practices and cultivating a “greener” ethos [12,13].

Marketers consistently deploy promotional strategies to encourage the purchase of ecological products [14]. According to Jäger and Weber [15], within ecological markets, green advertising is essential for publicizing a product’s features and advantages, thereby motivating consumers to choose environmentally protective options. Concurrently, eco-labels have emerged as a pivotal marketing instrument because they effectively inform consumers about the eco-friendly attributes of products [16,17]. Businesses are increasingly integrating eco-labels and green advertising into their communication tactics to facilitate the sale of eco-conscious goods [18,19]. Nevertheless, the consumer’s willingness to buy products marketed as environmentally friendly is often undermined by a perceived lack of authenticity and trust in such advertising [13]. Moreover, the term “environmental truth” is met with skepticism by consumers, leading to a diluted impact of green marketing efforts, as the endeavors of organizations can be overlooked [20].

Numerous studies have established that attitudes and environmental consciousness are key determinants in consumers’ intentions to buy green products [20,21]. According to Jaiswal and Kant [1], define environmental attitude as the consumer’s positive or negative evaluation that shapes the adoption of behaviors supportive of environmental protection. Conversely, environmental awareness represents a mindset that prompts consumers to select products that support the conservation of natural resources and ecosystems [22,23].

While numerous authors agree that attitudes and environmental awareness are predictive factors for green consumption, a literature review has highlighted gaps concerning the impact of these factors on purchasing behaviors. Riskos et al. [17] identified a notable discrepancy between environmental attitudes and the purchasing behavior of organic products, presenting a challenge for marketers to bridge. Malik et al. [24] noted a scarcity of evidence linking environmental awareness with green purchasing behavior. Conversely, consumers who identify with environmental protection tend to be more drawn to green products [2,3,5,6]. Yet, academic literature has not conclusively established whether the environmental attitudes and awareness of consumers stem from green advertising by companies or the presence of eco-labels on products. In light of this Song et al. [25] highlighted the need for research into how eco-labels might influence green purchasing behaviors, with a focus on attitude and environmental awareness as intermediary factors. Meanwhile Agarwal and Kumar [26] emphasized the importance of understanding whether green advertising effectively promotes the environmental awareness of consumers who purchase green products. 

The research problem centers on the paucity of studies that determine if green advertising and eco-labels act as catalysts for fostering environmental attitudes and enhancing consumer awareness of the significance of consuming organic products for environmental protection. In light of this, the pivotal research question being addressed is: Do green advertising and eco-labels shape the attitudes and environmental awareness of millennials frequently who buy organic products?

## 2. Literature Review

### 2.1. Green Purchasing Behaviour 

In recent decades, there has been a notable increase in consumers’ preference for food products characterized by ecological attributes and certifications [27]. The significance of understanding the motivations behind green purchasing behavior (GPB) has emerged as a prominent research area in the scientific community [7,20,21,28]. Scholars have also recognized the importance of exploring the factors that influence GPB among millennials [2,11,23,25]. Millennials, who are considered the largest consumer generation [20,29], comprise individuals born from 1979 to 2000 [12]. Their attitudes and buying patterns are distinguished by a preference for environmentally friendly products [6]. Furthermore, they typically take personal responsibility for environmental awareness issues [1]

### 2.2. Environmental Attitude 

Environmental attitude (EAT) reflects an individual’s positive valuation of behaviors that are aimed at minimizing negative impacts on the environment [22]. Research has shown that consumers with a positive disposition towards organic foods regard their purchase as significant and beneficial [20,21]. Kumar et al. [30] found that a favorable stance towards environmentally sustainable products bridges the gap between environmental knowledge and green purchasing behaviors. Jaiswal and Kant [1] have observed that both direct and indirect cognitive factors influence the likelihood of purchasing green products, facilitated by attitudes.

Taufique and Vaithianathan [22] concluded that EAT have a substantial effect on green purchasing and the behaviors of ecologically aware consumers. Recent studies also support a positive correlation between the attitudes towards green purchasing and the intent to purchase [2,20]. However, Sharma et al. [16] and S.h Ahmad et al. [31] point out an inconsistency between attitudes and actions; despite pro-environmental attitudes, consumer behavior does not always translate into the purchase of organic products. This disparity between EAT and green purchasing behavior (GPB) with respect to organic products presents a significant challenge for marketers [17]. Based on these findings, the following hypothesis has been formulated.

**Hypothesis** **1 (H1).** *Environmental attitudes influence the purchasing behaviors of millennials who consume organic products.*

### 2.3. Environmental Awareness 

Environmental awareness (EAW) is a cognitive construct that influences an individual’s concern for the environment and stimulates behavior that supports environmental protection [1,22,32]. Bülbül et al. [33] describe EAW as comprising two dimensions: (a) a sensitivity dimension, which acknowledges that consumers are alert to environmental issues, and (b) a willingness dimension, reflecting a readiness to purchase environmentally friendly products, despite potentially higher costs and limited availability.

Numerous studies have explored the connection between EAW and green consumption. For instance, Suárez et al. [34] examined EAW’s role in promoting environmental behavior and found that it does not invariably lead to personal actions that benefit the environment. Conversely, Shelest et al. [35] have identified EAW as a key predictor of pro-environmental behaviors. Aliman and Astina [36] suggest that EAW encourages individuals to adopt eco-protective behaviors. Current research underpins the impact of EAW on behaviors consistent with environmental conservation, demonstrating that increased EAW correlates with heightened concern for environmental issues [37,38]. This concern, in turn, leads to ecological behavior and a tendency to prefer environmentally friendly products [39]. While existing literature incorporates EAW into comprehensive models to ascertain its effect on green purchasing [1,22,37,39,40], some scholars advocate for more extensive research into this link, citing insufficient evidence on the relationship between EAW and GPB [24]. Given these considerations, the following hypothesis has been formulated.

**Hypothesis** **2 (H2).** *Environmental awareness influences the purchasing behaviors of millennials who consume organic products.*

### 2.4. Green Advertising

Green advertising (GAD) is the practice of crafting advertising messages that companies use to showcase the environmentally protective features of their products. Nguyen [41] notes that GAD focuses on broadcasting the ecological benefits and attributes that contribute to environmental preservation. Exposure to GAD influences consumers to form positive judgments and attitudes towards the environment, steering their purchasing choices towards products with minimal impact on the ecosystem [42,43]. Extensive research on green consumption has integrated GAD into their analytical frameworks, assessing its effect on consumers’ purchasing decisions [41]. Consequently, it has been suggested that GAD is positively associated with consumers’ intentions to purchase green products [13,44,45]. Nonetheless, some studies contradict this view, finding that GAD can engender negative brand perceptions and skepticism about the product’s touted environmental benefits [46]. Pittman et al. [47] have observed that the appeal of GAD can at times be regarded as deceptive. This skepticism, arising from green advertising, has been termed ‘greenwashing’ [48].

The literature review highlights the divergent views on GAD’s role in green consumption. Additionally, there appears to be a gap in understanding how GAD affects EAT, EAW, and GPB among millennials. Considering this and following Agarwal and Kumar’s [26] recommendation that further investigation is necessary to discern whether green advertising effectively promotes environmental awareness among consumers, the following hypotheses are proposed

**Hypothesis** **3 (H3).** *Green advertising influences the environmental attitude of millennials who consume organic products.*

**Hypothesis** **4 (H4).** *Green advertising influences the environmental awareness of millennials who consume organic products.*

**Hypothesis** **5 (H5).** *Green advertising directly influences the buying behaviors of millennials who consume organic products.*

### 2.5. Ecolabel

The eco-label (ECL) is a communicative tool that companies utilize to inform consumers about the environmentally friendly attributes of their products [41]. Panopoulus et al. [49] describe ECL as a strategic asset used by organizations to appeal to consumers who prefer products that minimize environmental harm. Contemporary studies have shown that informed consumer choices hinge on awareness of the environmental repercussions of their consumption. Hence, ECLs serve as primary information conduits about the ecological benefits of products [13,17,25,49,50,51]. In this context, ECL has evolved into a significant value proposition that businesses extend to their clientele [52].

While numerous studies on green purchasing confirm that ECLs sway consumers’ intentions towards environmentally responsible products [13,19,49,51], there is a noted scarcity in the literature examining the correlation between ECL, EAT, and EAW. Recognizing this gap, Song et al. [25] advocate for further research to understand the influence of ECL on EAT and EAW, and consequently on GPB. With these insights, the following hypotheses have been put forward.

**Hypothesis** **6 (H6).** *Eco-labels influence the environmental attitude of millennials who consume organic products.*

**Hypothesis** **7 (H7).** *Eco-labels influence the environmental awareness of millennials who consume organic products.*

**Hypothesis** **8 (H8).** *Eco-labels directly influence the purchasing behaviors of millennials who consume organic products.*

### 2.6. Conceptual Model

**Figure 1:**
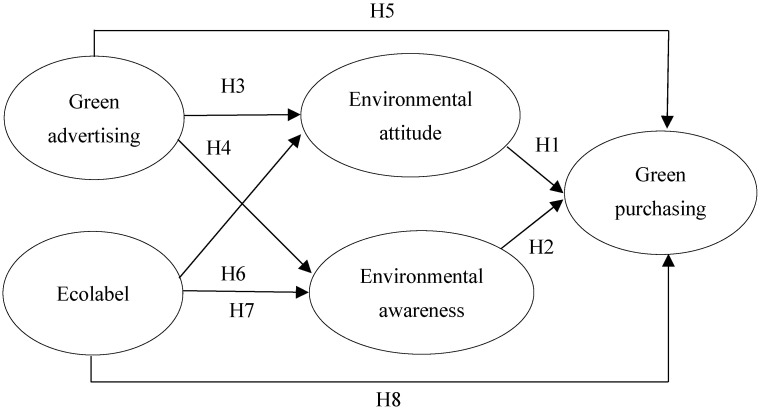
Research hypothesis model.

Building upon the insights provided by scholars about the aforementioned variables, the research model depicted in Figure 1 is designed to examine the impact of green advertising and eco-labeling on the environmental attitudes and awareness of millennials who buy organic products.

## 3. Materials and Methods

### 3.1. Instrument Design and Data Collection

This study employed a quantitative-correlational methodology with a cross-sectional design. Data were gathered through a self-administered written survey conducted in May 2023. The survey was administered in person to individuals outside shopping centers in Quito and Guayaquil, Ecuador. Employing probabilistic sampling methods, 430 millennials who self-identified as consumers of organic products willingly participated in the study. The inclusion criteria were limited to millennials who reported frequent purchases of organic products in the preceding week, while those who did not regularly consume organic products were excluded from the study.

The survey instrument was reviewed and validated by a panel consisting of two marketing experts and two research experts, who provided no amendments to the survey questions. Subsequently, a pilot test was conducted with 30 millennials to ascertain the relevance and comprehensibility of the questions. The survey comprised 20 questions derived from scholarly articles on green consumption, detailed further in Appendix A. The responses to the survey questions were measured using a five-point Likert scale.

### 3.2. Internal Consistency of the Instrument

After having applied the surveys of the study, it was necessary to determine the instrument’s internal consistency, for which the statistical procedures developed in recent studies on green consumption were applied [2,20]. The instrument’s internal consistency was initially tested through a Cronbach’s alpha test. The consistency analysis determined that discarding four questions (EAT4, EAW1, GAD1, ECL2) was necessary, leaving 16 questions for statistical analysis. Then, the Cronbach’s alpha test of the instrument was calculated again, and the result was 0.824. 

### 3.3. Data Analysis

A Confirmatory Factor Analysis (CFA) was performed to measure the convergent and discriminant validity of the variables of the hypothesized model. Regarding the convergent validity, the factorial loads of the indicator variables were calculated, followed by the Composite Reliability (CR) and the Average Variance Extracted (AVE) of the model constructs. For the discriminant validity, the Square Root of the AVEs (SRAVE) was compared with the values of the correlations of the constructs. Excel 17.0 (2019)and SPSS 24 were used to calculate these values. The acceptance or rejection of the hypotheses was determined through the implementation of structural equation models. Multiple indices were used to ensure the fit of the model. Such as the relative value of x^2^ of the degree of freedom (x^2^/gL), the Goodness of Fit Index (GFI), the Comparative Fit Index (CFI), the Tucker-Lewis index (TLI), and the Normalized Fit Index (NFI). Finally, the Mean square residue (MSR) and the Mean Square Error of Approximation (MSEA) were calculated. AMOS 24 program was used to calculate these values.

## 4. Results

### 4.1. Demographic Characteristic of Respondents

The study was conducted in the Ecuadorian cities of Quito and Guayaquil. Out of 465 millennials approached, 430 agreed to participate, resulting in an acceptance rate of 92%. Carrión et al. [20] suggest that a sample is considered adequate if it includes at least 20 respondents for each survey question. Consequently, this study required a minimum of 400 respondents. Table 1 presents the demographic characteristics of the participants in the study.

### 4.2. Estimation of the Measurement Model

The hypothesized model, composed of five variables (environmental attitude, environmental awareness, green purchasing behavior, green advertising, and ecolabels), was tested using a CFA. It was necessary to determine the reliability and convergent validity through values of Cronbach’s alpha ≥ 0.70, CR ≥ 0.70, and AVE ≥ 0.50 [2]. When Cronbach’s alpha values exceed ≥ 0.70, CR values are ≥0.7, and AVE values are ≥0.50 and lower than AVE values, convergent validity can be confirmed [20]. See Table 2.

To establish discriminant validity, it was necessary to compare the square roots of the Average Variance Extracted (AVE) values for each construct with the correlation coefficients of each construct pair within the model. Discriminant validity is confirmed when the square roots of the AVE (SR AVE) are greater than the correlations between each pair of constructs [20]. See Table 3.

After evaluating the convergent and discriminant validity criteria of the research model, a Structural Equation Modeling (SEM) analysis was conducted to ascertain the acceptance or rejection of the hypotheses. This analysis examined the interrelationships among the five variables specified in the proposed model.

**Figure 2:**
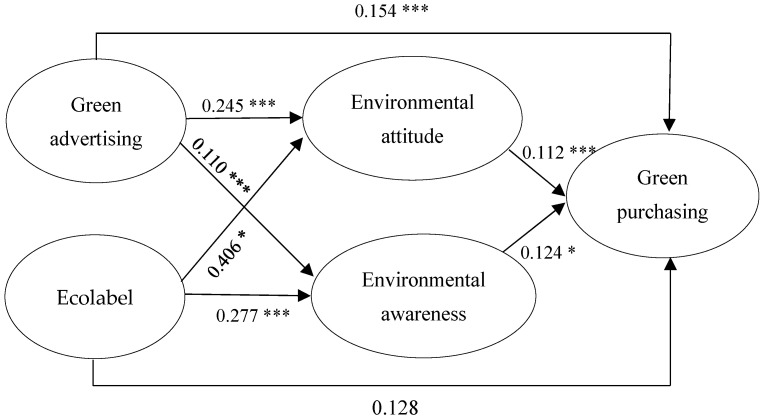
Beta values and significance levels that explain the acceptance and rejection of the relationships tested in the hypothesized model. *** Correlation is significant at 0.01 (bilateral), * Correlation is significant at 0.05 (bilateral).

The results determined by the maximum likelihood estimate showed that the data met the goodness of fi t indices: x^2^ (df) = 132.704 (96); x^2^/g = 1.382; NFI = 0.978; TLI = 0.992; CFI = 0.944; root mean square error of approximation (RMSEA) = 0.030. After examining the relationships between the five variables of the hypothesized model, seven hypotheses were accepted, and one was rejected. The estimated values obtained through AMOS 24 allowed us to determine that EAT (β = 0.112 *p* < 0.001), and EAW (β = 0.124 *p* < 0.005), influence GPB. Likewise, it was determined that GAD influences EAT (β = 0.245 *p* < 0.001), also influences EAW (β = 0.110 *p* < 0.001), and also directly influences GPB (β = 0.154 *p* < 0.001). While ECL influences EAT (β = 0.406 *p* < 0.005), they also influence EAW (β = 0.277 *p* < 0.001) but do not directly influence GPB (β =0.128 *p* > 0.005). See Table 4 and Figure 2.

## 5. Discussion

The literature review indicates a limited amount of research on green consumption within the context of Ecuador. Thus, this study is among the first to explore the connection between green advertising elements and the green purchasing behaviors of Ecuadorian millennials. The results from the Structural Equation Modeling (SEM) enabled the acceptance of seven hypotheses posited in the model while leading to the rejection of one. 

Hypothesis 1 is confirmed, suggesting that EAT positively influences the GPB of millennials who frequently consume organic products. This result demonstrates a significant link between EAT and the purchasing behavior for organic products, illustrating that millennials are environmentally concerned [11,12,21,29] and feel a sense of responsibility towards environmental protection [3,6,23,25,29]. This sense of responsibility motivates them to be a demographic that not only engages in pro-environmental actions but also promotes such consumption behaviors among their peers [2,11,36]. This finding aligns with the conclusions of several researchers who have identified that attitude is a crucial determinant of both the intention to purchase and the actual purchasing behavior for organic products [1,2,4,11,20,22,27,32,53,54], and it is closely associated with GPB [3,7]. Conversely, it challenges the assertions of previous studies which cast doubt on the influential role of attitude in GPB [17], particularly those positing an attitude-behavior gap where consumers with EAT do not consistently purchase organic products [16,31], highlighting discrepancies between their stated beliefs and actions [2,3,4,5,6,7,8,9,10,11,12,13,14,15,16,17,18,19,20]. Consequently, this result confirms that EAT continues to significantly impact millennials’ GPB, offering insights to researchers who have noted gaps between attitudes and behaviors [7]. It is important to note, however, that not all environmentally conscious millennials need to consume organic products to make a positive impact; some contribute to environmental protection through actions like recycling [41].

Hypothesis 2 is accepted, indicating that EAW positively influences the GPB of millennials who frequently consume organic products. The results show that EAW is a positive evaluation of the behaviors one should adopt to protect and support the environment [6,11,21,22,27], suggesting that millennials believe humanity is exploiting nature and causing disastrous environmental consequences [21,35,36]. While this finding challenges research such as the study by Suárez et al. [34], which argued that EAW does not always result in personal actions to preserve the environment, it supports other studies demonstrating that millennials are concerned about environmental issues, leading to increased EAW [2,11,20,23,29,37]. Furthermore, it is evident that the purchasing behaviors of this demographic are influenced by their level of awareness and commitment to responsible consumption [1,30,34,35,36,37,39]. Therefore, this confirms that EAW is a determinant factor in GPB, addressing the concerns of scholars who have noted a lack of evidence regarding the impact of EAW on GPB and have called for further investigation of this relationship [24].

Hypothesis H3 posits that GAD influences the millennial generation’s consumption of green products, and it is accepted. This result supports the notion that millennials who frequently consume organic products pay particular attention to companies’ advertising messages that highlight the eco-friendly features of organic products [48,55,56]. It confirms that consumers exposed to GAD form positive attitudes toward the environment [42,43]. Additionally, this finding aligns with other studies that have identified a connection between GAD and EAT, suggesting that advertising messages can foster attitudes conducive to the consumption of environmentally friendly products [48,57,58]. Conversely, this study’s findings challenge the work of some researchers who argue that advertising does not influence all organic product consumers equally [13]. Some consumers may not respond positively to GAD, perceiving it instead as greenwashing [59,60,61,62], indicating that EAT may be driven by factors other than GAD [48].

Similarly, Hypothesis H4 is accepted, confirming that GAD influences the EAW of millennials who frequently consume organic products. This substantiates that GAD effectively disseminates an ecological image of products and, through brand positioning, boosts consumer awareness [63,64]. It supports the notion that GAD heightens consumer consciousness and stimulates their environmental awareness, thus encouraging the purchase of organic products [48,50]. This finding is in line with other research that has established a positive relationship between GAD and EAW [37,38,55,56,65,66] and challenges those studies which assert that GAD does not enhance the EAW of organic product consumers [13,61].

Furthermore, the study confirmed a direct link between GAD and GPB; therefore, Hypothesis H5 is accepted, meaning that GAD directly affects the GPB of millennials who frequently consume green products. In other words, GAD remains a pivotal marketing element that promotes the consumption of environmentally protective products [42]. This finding corroborates substantial evidence of the direct impact of this marketing strategy on environmentally conscious purchasing behavior [43,50,55,63] and supports the conclusions of other research highlighting the significant role of GAD in influencing GPB [40,44,48,56,64]. It also addresses the skepticism of researchers who continue to question the role of GAD within organic purchasing behavior [46,47,48,53].

The validation of Hypotheses H3, H4, and H5 confirms the effect of GAD on EAT, EAW, and GPB among millennials who frequently buy organic products. These results bridge the knowledge gap created by the ongoing debate among scholars regarding the influence of GAD on GPB. They also provide insights in response to Agarwal and Kumar [26], who pointed out the need to explore whether GAD fosters EAW among consumers.

Hypothesis H6 is confirmed, suggesting that eco-labels (ECL) influence the EAT of millennials who frequently consume organic products. The results indicate that millennials view ECL as an effective means of informing consumers about the ecological attributes of organic products, thereby enhancing their trust and encouraging the purchase of such products [41,51]. This outcome aligns with findings from various studies which have shown that ECL reduce consumer skepticism [25] and bolster their favorable attitudes towards the consumption of organic products [13,17,25,49,50]. The result also corroborates research identifying ECL as informative tools about the ecological aspects of products [3,41,49,51], particularly those establishing a link between ECL and EAT [17]. Conversely, it challenges studies that have found ECL does not significantly influence consumer attitudes [67].

Hypothesis H7 is confirmed, showing that ECL affect the EAW of millennials who frequently consume organic products. This confirms that millennials believe products with ECL demonstrate a commitment to the environment, a belief that significantly shapes their EAW [13]. They trust that the attributes listed on the product’s eco-label are genuine [67,68], leading to satisfaction with their purchase and the reassurance that their consumption habits are environmentally benign [18,19,41,42,43,44,45,46,47,48,49,50,51]. This finding supports research that has demonstrated the impact of ECL on EAW [13,25,56] and challenges studies that have cast doubt on the connection between these two variables [68].

Finally, Hypothesis H8 is rejected, indicating that ECL do not directly influence GPB. This suggests that ECL alone are not a determining factor in the frequent purchase of organic products by millennials. This conclusion is at odds with several studies that have found ECL to be a significant influence on GPB [3,13,17,25,49,50,56,67]. It has been demonstrated, however, that while ECL can enhance the attitudes and awareness of organic product consumers [18,19,41,51], they do not have a direct impact on purchasing behaviors.

Although the study resulted in the acceptance of H6 and H7 and the rejection of H8, these outcomes underscore the impact of ECL on the purchasing behaviors of organic products among millennials. This provides an answer to Song et al. [25], who suggested the need for research into how ECL affects EAT and EAW, and subsequently, how it influences GPB.

The current study has practical, theoretical, and social implications. Practically, the findings enable organic food companies to recognize the significance of GAD in the brand positioning of a product, as well as the importance of fostering pro-environmental attitudes and enhancing consumer EAW. Furthermore, this research provides insights for organic food producers on the value of ECL within their product strategies. Beyond visual appeal, ECL serves as a means for consumers to understand the environmental attributes of a product. Theoretically, the results contribute empirical knowledge that both supports and challenges prior research, thus broadening the domain of green consumption knowledge and enhancing our understanding of the green purchasing behaviors of Ecuadorian consumers. Socially, the research confirms that millennials are mindful of environmental issues and are inclined to support sustainability through their consumption choices. Consequently, there is an appeal for government bodies to initiate policies that benefit small-scale producers, encouraging them to engage in environmentally friendly production and develop organic products for improved consumer health.

This study has three limitations. Firstly, it focused solely on millennials, excluding other demographic groups like Generation X, who have significant purchasing power and familiarity with traditional advertising, and thus may also be influenced by GAD. Secondly, advertising is merely one among many marketing tools that can affect consumer behavior, and this study did not account for other influential mediums, such as social media and sales promotions, which could also affect EAT and EAW. Lastly, the sample was drawn from just two cities in Ecuador, limiting the generalizability of the findings to the entire Ecuadorian populace.

To address these limitations, future research should conduct comparative studies among different demographic groups, including centennials, millennials, Generation X, and Baby Boomers, to determine which group is most aligned with environmental protection and green consumption. Additionally, it is crucial to expand upon the research model used in this study to explore whether other marketing strategies, such as branding, ecological packaging, sales promotions, or social media, influence environmentally conscious consumer attitudes. Finally, subsequent research could broaden the sampling frame to include millennials from other Ecuadorian cities and contrast these findings with those from millennials in different regions.

## 6. Conclusions

Research into organic consumption has become increasingly significant within academic circles, with numerous studies aimed at identifying the factors that influence environmentally identified product purchasing behaviors. While the impact of EAT and EAW on organic product behaviors is well-documented, evidence is scant regarding the influence of ECL and GAD on the attitudes and awareness of millennials who frequently consume organic products, particularly in developing countries and in South America. This study demonstrates that Ecuadorian millennials hold favorable attitudes toward the environment and their levels of EAW are in line with environmental concerns, which in turn directly influence their organic product purchasing behaviors.

The study addressed the research question, “Do GAD and ECL influence the EAT and EAW of millennials who consume frequently organic products?” and determined that: (a) GAD influences EAT and EAW, which then affect GPB, and GAD also directly influences the GPB of Ecuadorian millennials, and (b) ECLs impacts EAT and EAW, which subsequently affect GPB, although ECLs does not directly influence the GPB of Ecuadorian millennials.

## Figures and Tables

**Table 1:** Demographic characteristics of study participants.

Characteristics	Category	n	%
City	Quito	218	51
	Guayaquil	212	49
Education level	Postgraduate	155	36
	Undergraduate	275	64
Millennial cohort	Older Millennials (35 to 44 years old)	185	43
	Mid Millennials (29 to 34 years old)	130	30
	Younger Millennials (23 to 28 years old)	115	27
Gender	Male	247	57
	Female	183	43
n = 430			

**Table 2:** Convergente validity and reliability.

Variable	Item	Loading Factor	Cronbach Alpha	CR	AVE
Environmental attitude (EAT)	EAT1	0.955	0.943	0.948	0,86
	EAT2	0.916			
	EAT3	0.911			
Environmental awareness (EAW)	EAW2	0.945	0.932	0.938	0.835
	EAW3	0.871			
	EAW4	0.924			
Green purchasing behaviour (GPB)	GPB1	0.696	0.857	0.889	0.670
	GPB2	0.911			
	GPB3	0.730			
	GPB4	0.913			
Green advertising (GAD)	GAD2	0.906	0.881	0.894	0.739
	GAD3	0.783			
	GAD4	0.884			
Eco-labels (ECL)	ECL1	0.866	0.825	0.864	0.680
	ECL3	0.862			
	ECL4	0.739			
**Alfa total**	**0.824**				

**Table 3:** Reliability and validity.

	F1	F2	F3	F4	F5	SR AVE
F1	**0.860 ^a^**					0.927
F2	0.135 **	**0.835 ^a^**				0.913
F3	0.238 **	0.173 **	**0.670 ^a^**			0.818
F4	0.220 **	0.193 **	0.291 **	**0.739 ^a^**		0.859
F5	0.105 *	0.224 **	0.137 **	0.048 *	**0.680 ^a^**	0.824

**^a^** AVE, ** Correlation is significant at 0.01 (bilateral), * Correlation is significant at 0.05 (bilateral). Note: F1: Environmental attitude, F2: Environmental awareness, F3: Green purchasing; F4: Green advertising; F5: Ecolabel. F1-F3; F2-F3; F4-F1; F4-F2; F4-F3; F5-F2, and F5-F3 had significant correlation at bilateral level 0.01. F5-F1 had a significant correlation at bilateral level 0.05.

**Table 4:** Results of hypotheses testing.

Hypothesis	Relation	*β*	*p*-Values	Hypothesis
H1	EAT-GPB	0.112	***	Accepted
H2	EAW-GPB	0.124	0.039 *	Accepted
H3	GAD-EAT	0.245	***	Accepted
H4	GAD-EAW	0.110	***	Accepted
H5	GAD-GPB	0.154	***	Accepted
H6	ECL-EAT	0.406	0.010 *	Accepted
H7	ECL-EAW	0.277	***	Accepted
H8	ECL-GPB	0.128	0.190	Rejected

*** Correlation is significant at 0.01 (bilateral). * Correlation is significant at 0.05 (bilateral).

## Data Availability

Survey, statistical data and analysis of the research available at: https://drive.google.com/drive/folders/1N5lYNAiwx_Q4Uo3vkYDxvquLgIOaesLN?usp=sharing, (accessed on 18 December 2023).

## Appendix A

**Table A1:** Survey qestions.

Variable	Item	Answer Option
Environmental attitude [54]	EAT1	I am very concerned about the environment
	EAT2	I am willing to reduce my consumption to help the environment.
	EAT3	I would contribute financially to help protect the environment.
	EAT4	I have asked my family to recycle some of the things we use.
Environmental awareness [54]	EAW1	I believe that humanity is seriously abusing the environment.
	EAW2	I think that humans produce disastrous consequences in nature.
	EZW3	I consider that the balance of nature is very delicate and easily upset.
	EAW4	I think that one must live in harmony with nature in order to survive.
Green purchasing behaviour [20]	GPB1	I buy organic products regularly.
	GPB2	I buy organic products for my daily needs.
	GPB3	I have bought organic products for the last few months.
	GPB4	I buy organic products, although there are conventional alternatives.
Green advertising [13]	GAD1	I tend to focus on advertising messages that relate to the environment.
	GAD2	I think brands that use advertising messages about the environment are good.
	GAD3	I pay attention to products that develop advertisements that relate to the environment.
	GAD4	I find green advertising valuable in my opinion.
Ecolabels [17,41]	ECL1	I consider the eco-labels displayed on the product to be a good way to inform consumers.
	ECL2	I believe that eco-labelled products meet reliable environmental quality standards.
	ECL3	The presence of certified organic labels increases my credibility in a product.
	ECL4	I believe that eco-labeled products are really committed to protecting the environment.
